# Supplementary material for: Trophic ecology outweighed intrinsic constraints in shaping skull evolution of carnivorous Permian synapsids
Source: Commun Biol. 2026 Mar 12;9:588. doi: 10.1038/s42003-026-09824-3 (PMC13125638; doi:10.1038/s42003-026-09824-3)
Supplement: Supplementary file 2 — Supplementary Information [file 42003_2026_9824_MOESM2_ESM.pdf]

Supplementary Information for

Trophic ecology outweighed intrinsic constraints in shaping skull evolution of carnivorous Permian synapsids

Elías Adán Warshaw* *et al.*
*Corresponding author. Email: elias.warshaw.25@ucl.ac.uk

This PDF file includes:

Supplementary Figures 1 to 3
Supplementary Table 1

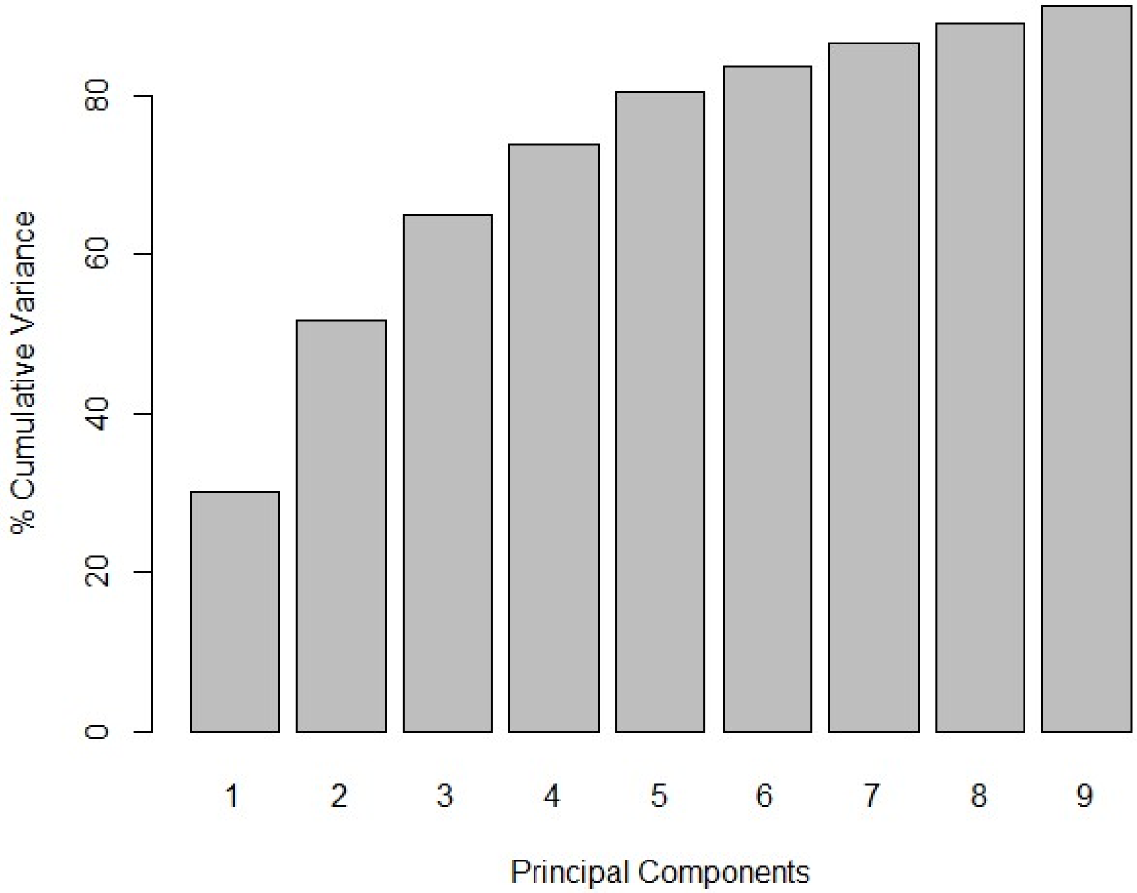

Supplementary Figure 1.
Proportion of variance encapsulated within shape PCs. Note change in slope after PC5.

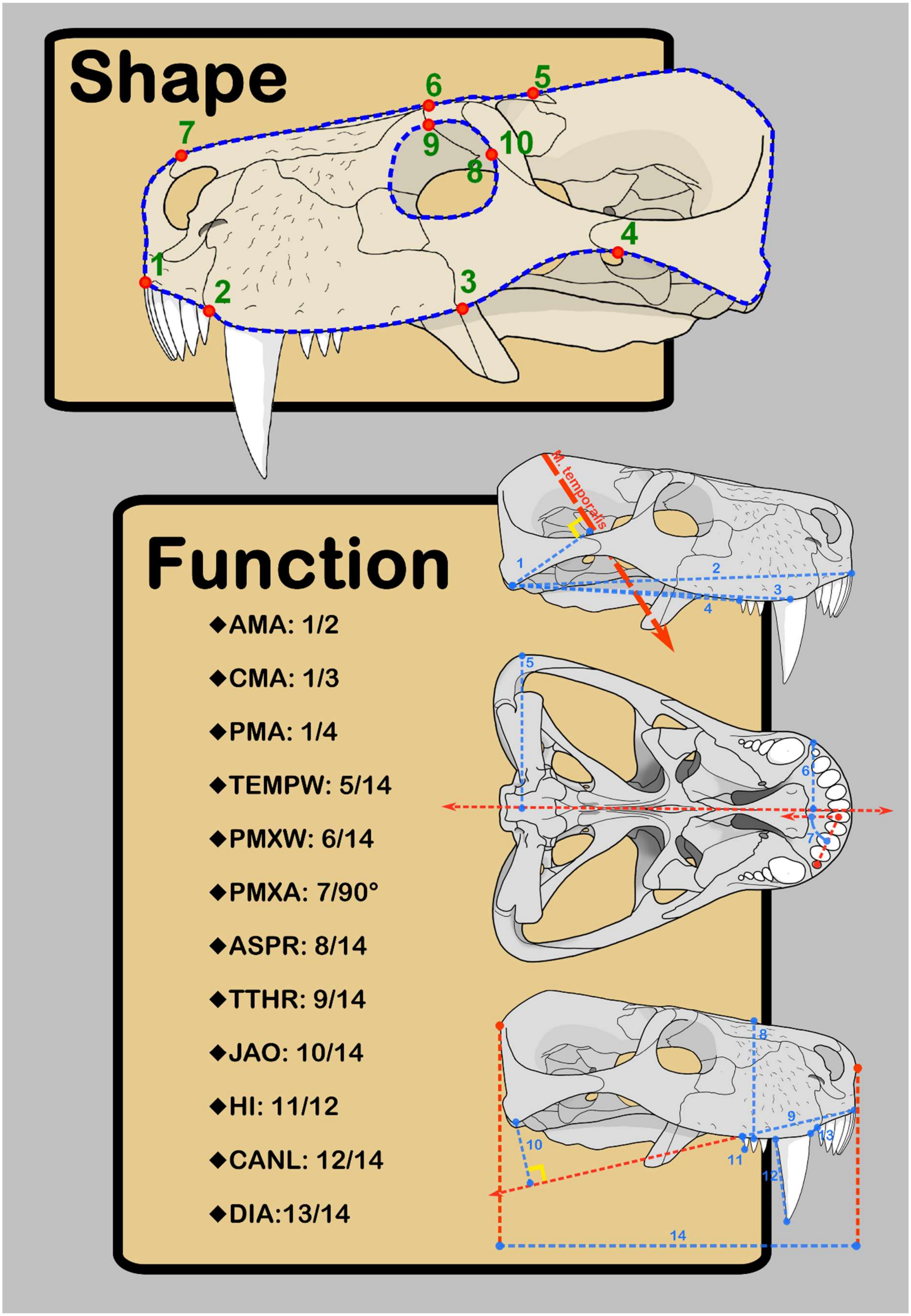
Shape
1
2
3
4
5
6
7
8
9
10
Function
◆AMA: 1/2
◆CMA: 1/3
◆PMA: 1/4
◆TEMPW: 5/14
◆PMXW: 6/14
◆PMXA: 7/90°
◆ASPR: 8/14
◆TTHR: 9/14
◆JAO: 10/14
◆HI: 11/12
◆CANL: 12/14
◆DIA:13/14
M. temporalis

Supplementary Figure 2.

Landmarking regime and functional morphometric measurements. Shape landmarks are as follows: **1**. Anterior-most point of the premaxilla along the alveolar margin; **2**. Anterior-most point of the maxilla along the alveolar margin; **3**. Posterior-most point of the maxilla along its ventral margin; **4**. Anterior-most extent of the squamosal along the ventral margin of the skull; **5**. Anterior-most extent of the squamosal along the caudodorsolateral margin of the skull; **6**. Point on the skull roof directly dorsal to the caudalmost extent of the prefrontal along the orbital margin; **7**. Contact between the nasal and premaxilla along the rostrodorsal margin of the skull; **8**. Posterodorsal-most extent of the jugal along the caudoventral margin of the orbit; **9**. Posterior-most extent of the prefrontal along the dorsal margin of the orbit; **10**. Posteroventral-most extent of the postorbital along the posteroventral margin of the orbit; **11-13**. Three semilandmarks between landmarks 1 and 2; **14-28**. Fifteen semilandmarks between landmarks 2 and 3; **29-43**. Fifteen semilandmarks between landmarks 3 and 4; **44-63**. Twenty semilandmarks between landmarks 4 and 5; **64-78**. Fifteen semilandmarks between landmarks 5 and 6; **79-88**. Ten semilandmarks between landmarks 6 and 7; **89-93**. Five semilandmarks between landmarks 7 and 1; **94-108**. Fifteen semilandmarks between landmarks 8 and 9; **109-123**. Fifteen semilandmarks between landmarks 9 and 10. Equations for functional characteristics refer to numbered measurements at right; see main article text for abbreviations. Depicted skull is a composite specimen of *Moschorhinus kitchingi*; see Fig. 1 in main article text for source.

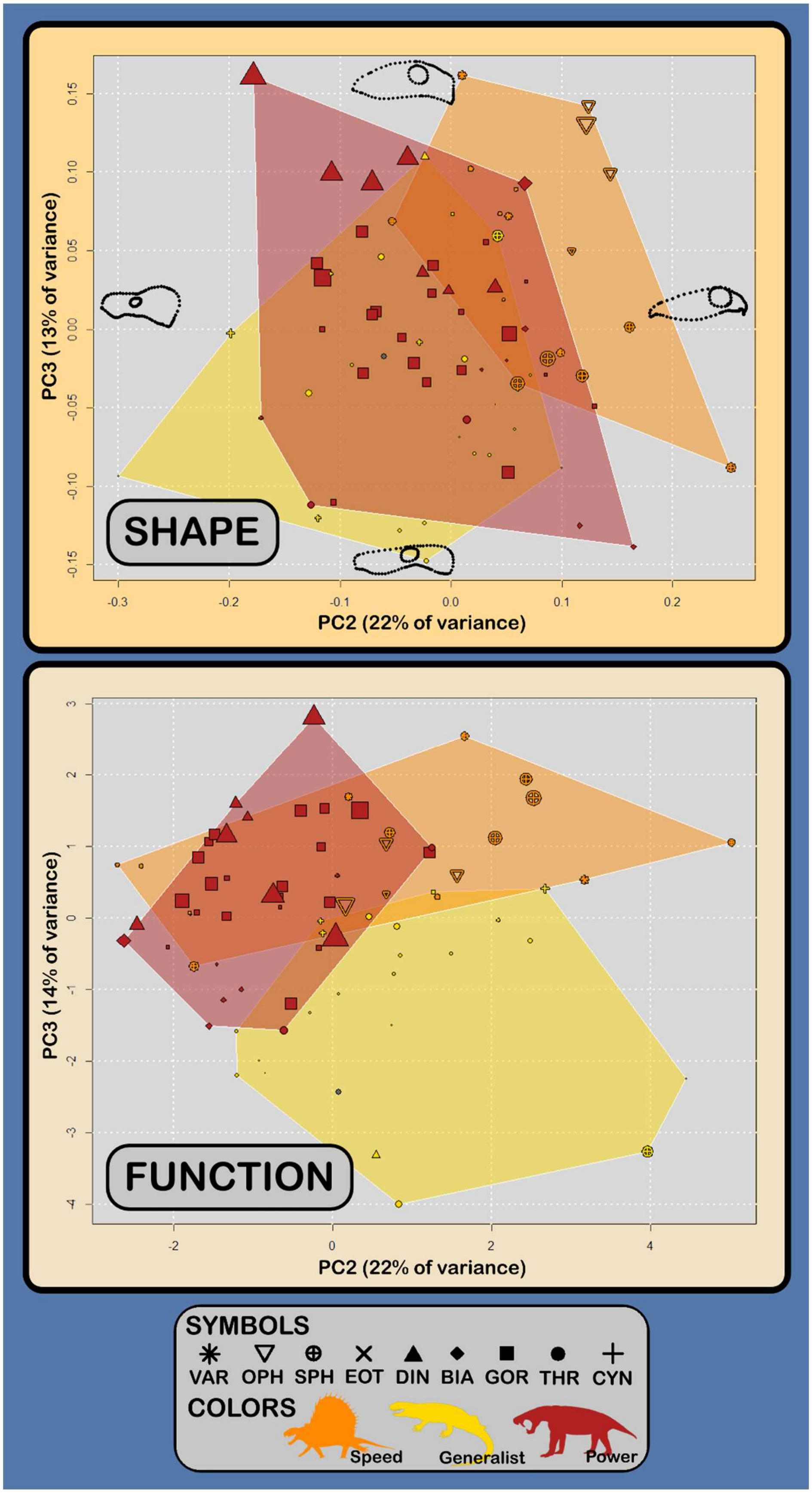
SHAPE
PC2 (22% of variance)
PC3 (13% of variance)
FUNCTION
PC2 (22% of variance)
PC3 (14% of variance)
SYMBOLS
VAR OPH SPH EOT DIN BIA GOR THR CYN
COLORS
Speed
Generalist
Power

Supplementary Figure 3.

Supplemental results of PCA on shape and function. Plots show PCs 2 and 3 to supplement PC1/2 plots in main article text (n = 77 specimens). Skull outlines on shape PC axes show shapes corresponding to minimum and maximum PC values. Points are colored by FFG designation and are sized proportionally to centroid size. Abbreviations are as in man text figures. *Dimetrodon* and *Eothyris* silhouettes from phylopic courtesy of Scott Hartman and Nobu Tamura, respectively.

	Brownian motion	Early burst	Delta	Kappa
lnL	721.250	726.158	729.474	**736.891**
AIC	-1438.501	-1446.316	-1452.948	**-1467.783**

Supplementary Table 1.

Fit of different evolutionary models to functional data with only complete specimens (n=33). Best performing model is bolded. Abbreviations: AIC- Akaike Information Criterion; lnL – log-likelihood.
