# Supplementary material for: Trophic ecology outweighed intrinsic constraints in shaping skull evolution of carnivorous Permian synapsids
Source: Commun Biol. 2026 Mar 12;9:588. doi: 10.1038/s42003-026-09824-3 (PMC13125638; doi:10.1038/s42003-026-09824-3)
Supplement: Supplementary file 3 — Reporting summary [file 42003_2026_9824_MOESM3_ESM.pdf]

nature portfolio

Corresponding author(s): Elias A. Warshaw

Last updated by author(s): Jan 26, 2026

Reporting Summary

Nature Portfolio wishes to improve the reproducibility of the work that we publish. This form provides structure for consistency and transparency in reporting. For further information on Nature Portfolio policies, see our Editorial Policies and the Editorial Policy Checklist.

Statistics

For all statistical analyses, confirm that the following items are present in the figure legend, table legend, main text, or Methods section.

n/a	Confirmed	
☐	☒	The exact sample size (n) for each experimental group/condition, given as a discrete number and unit of measurement
☐	☒	A statement on whether measurements were taken from distinct samples or whether the same sample was measured repeatedly
☐	☒	The statistical test(s) used AND whether they are one- or two-sided<br>*Only common tests should be described solely by name; describe more complex techniques in the Methods section.*
☐	☒	A description of all covariates tested
☐	☒	A description of any assumptions or corrections, such as tests of normality and adjustment for multiple comparisons
☐	☒	A full description of the statistical parameters including central tendency (e.g. means) or other basic estimates (e.g. regression coefficient) AND variation (e.g. standard deviation) or associated estimates of uncertainty (e.g. confidence intervals)
☐	☒	For null hypothesis testing, the test statistic (e.g. F, t, r) with confidence intervals, effect sizes, degrees of freedom and P value noted<br>*Give P values as exact values whenever suitable.*
☒	☐	For Bayesian analysis, information on the choice of priors and Markov chain Monte Carlo settings
☒	☐	For hierarchical and complex designs, identification of the appropriate level for tests and full reporting of outcomes
☐	☒	Estimates of effect sizes (e.g. Cohen's d, Pearson's r), indicating how they were calculated

Our web collection on statistics for biologists contains articles on many of the points above.

Software and code

Policy information about availability of computer code

Data collection	Geometric morphometric data was collected with the open source software tpsDig and tpsUtil, which are cited in the manuscript. Linear morphometric data was collected with the open source software ImageJ. Anatomical network data was collected in Excel from Microsoft Office.
Data analysis	All analyses were carried out in R 4.3.0 using open source code, the sources of which are cited in the Methods section. All code is provided as a supplement alongside the formatted data necessary to run it.

For manuscripts utilizing custom algorithms or software that are central to the research but not yet described in published literature, software must be made available to editors and reviewers. We strongly encourage code deposition in a community repository (e.g. GitHub). See the Nature Portfolio guidelines for submitting code & software for further information.

Data

Policy information about availability of data

All manuscripts must include a data availability statement. This statement should provide the following information, where applicable:

- Accession codes, unique identifiers, or web links for publicly available datasets
- A description of any restrictions on data availability
- For clinical datasets or third party data, please ensure that the statement adheres to our policy

All raw data, including measurements, the specimens from which they were taken, and the published sources for photographs or reconstructions used to collect data, are available in the supplementary materials.

Research involving human participants, their data, or biological material

Policy information about studies with human participants or human data. See also policy information about sex, gender (identity/presentation), and sexual orientation and race, ethnicity and racism.

Reporting on sex and gender	N/A
Reporting on race, ethnicity, or other socially relevant groupings	N/A
Population characteristics	N/A
Recruitment	N/A
Ethics oversight	N/A

Note that full information on the approval of the study protocol must also be provided in the manuscript.

Field-specific reporting

Please select the one below that is the best fit for your research. If you are not sure, read the appropriate sections before making your selection.

☐ Life sciences ☐ Behavioural & social sciences ☒ Ecological, evolutionary & environmental sciences

For a reference copy of the document with all sections, see nature.com/documents/nr-reporting-summary-flat.pdf

Ecological, evolutionary & environmental sciences study design

All studies must disclose on these points even when the disclosure is negative.

Study description	Three separate sets of analyses were run: 1) Anatomical network analysis, using clustering algorithms to quantify modularity in the distribution of osseous contacts in the skulls and jaws of sampled specimens (n=8) as recorded by an adjacency matrix. 2) A geometric morphometric analysis, involving generalized procrustes analysis (GPA) followed by principal component analysis (PCA) to summarize shape variation across sampled specimens (n=77). 3) Consensus clustering analysis of linear morphometric data from the same dataset as the geometric morphometric analysis, using three different clustering algorithms to identify objective groupings of taxa according to variation in trait values related to trophic ecology. Data from each of these analyses were then compared using phylogenetic comparative methods, including evolutionary model fitting of shape PC data and linear morphometric data, and phylogenetically corrected regressions of functional measurements against shape data.
Research sample	Our sample included carnivorous synapsids from across the entire Permian period as well as the late Carboniferous, intended to be as comprehensive as possible with limits on sample size imposed by 1) existence and preservation quality of relevant specimens, 2) prior inclusion of taxa within a published phylogenetic analysis, providing phylogenetic context crucial to comparative methods, and 3) presence of relevant specimens within the published literature, from which all samples were taken.
Sampling strategy	Sample size represents the largest possible sample given constraints listed above.
Data collection	Samples were collected directly from the literature, with relevant articles identified using google scholar or physical media in some cases. Photos or reconstructions for morphometric analyses were collected by screenshotting published figures (after scanning them into pdf format, for physical sources), and anatomical network data was recorded within adjacency matrices in Excel (a matrix with rows and columns mirroring each other and representing individual bones, filled by denoting contacts between bones as "1").
Timing and spatial scale	Samples range from the late Carboniferous through the entire Permian period, representing the entire temporal range of the radiations under study. The sample is global, with any geographic heterogeneity being the result of either 1) biases in the fossil record or 2) differences in research productivity between different regions impacting the likelihood of specimens being published.

Data exclusions	No data were excluded following data collection. Taxa were excluded from the study as a whole if known only from incomplete, severely taphonomically deformed, or juvenile specimens in order to maximise the quality of the dataset and reduce extraneous sources of variation (e.g., ontogenetic, taphonomic).
Reproducibility	The sources for all data are cited in the supplementary materials, and all raw data is provided as well. All attempts to repeat experiments were successful.
Randomization	When analyses were repeated for subsets of the total dataset, groupings of taxa were purely phylogenetic, representing independent clades (or paraphyletic grades representing discrete radiations, e.g. basal synapsids). When necessary, covariates were corrected for the influence of phylogeny on trait values.
Blinding	Blinding was not relevant to our study, as we did not have randomized groupings or any analyses that required them.

Did the study involve field work? ☐ Yes ☒ No

Reporting for specific materials, systems and methods

We require information from authors about some types of materials, experimental systems and methods used in many studies. Here, indicate whether each material, system or method listed is relevant to your study. If you are not sure if a list item applies to your research, read the appropriate section before selecting a response.

Materials & experimental systems

n/a	Involved in the study
☒	☐ Antibodies
☒	☐ Eukaryotic cell lines
☐	☒ Palaeontology and archaeology
☒	☐ Animals and other organisms
☒	☐ Clinical data
☒	☐ Dual use research of concern
☒	☐ Plants

Methods

n/a	Involved in the study
☒	☐ ChIP-seq
☒	☐ Flow cytometry
☒	☐ MRI-based neuroimaging

Palaeontology and Archaeology

Specimen provenance	No new specimens are reported within our study, and we were not involved in the collection or initial study of any specimens included in our analyses. Our research is based on already published descriptions and/or photographs/illustrations of specimens.
Specimen deposition	All specimens included in our study are accessioned into reputable collections and are present within the literature. No new specimens are reported within our study.
Dating methods	No new dates are provided.

☒ Tick this box to confirm that the raw and calibrated dates are available in the paper or in Supplementary Information.

Ethics oversight	No ethical approval was necessary, as our study did not include the collection of any new specimens or any destructive sampling.

Note that full information on the approval of the study protocol must also be provided in the manuscript.

Plants

Seed stocks	N/A
Novel plant genotypes	N/A
Authentication	N/A
